# EHMTI-0050. Systemic and cerebral endothelial function in migraine

**DOI:** 10.1186/1129-2377-15-S1-F32

**Published:** 2014-09-18

**Authors:** M Zaletel, J Oblak Pretnar, B Zvan, D Perko

**Affiliations:** 1 Department of Vasular Neurology, University Clinical centre Ljubljana, Ljubljana, Slovenia

## Background

We showed different endothelial functions of the anterior and posterior cerebral circulation in healthy subjects, worse vasodilatatory capacity of the posterior cerebral circulation and unimpaired systemic endothelial function in migraine patients without comorbidities. The relationship between cerebral and systemic endothelial function and the anterior and posterior cerebral endothelial function in migraine patients is still not clear.

## Methods

We compared cerebral and systemic endothelial function through post-hoc linear regression analysis of cerebrovascular reactivity (CVR) to L-arginine between the middle cerebral artery (MCA) and flow mediated vasodilatation (FMD) of the right brachial artery and posterior cerebral artery (PCA) and FMD in migraine patients without comorbidities and in healthy subjects.

## Results

We did not find any significant correlation between CVR to L-arginine in the MCA and FMD and PCA and FMD in migraine patients with aura (p = 0.880 vs. p = 0.682), without aura (p = 0.153 vs. p = 0.179) and healthy subjects (p = 0.869 vs. p = 0.662). On the other hand we found a significant correlation in CVR to L-arginine between the MCA and PCA in migraine patients with aura (p = 0.004), without aura (p = 0.001) and in healthy subjects (p = 0.002).

## Conclusions

Our study suggests that the endothelial function of cerebral and systemic circulation might be different in migraine patients without comorbidities, while that of the anterior and posterior cerebral circulation might be coupled with a worse vasodilatatory capacity in the posterior cerebral circulation, which could indicate endothelial dysfunction in this territory.

No conflict of interest.

